# A metagenomic survey reveals widespread antibiotic resistance genes in honey bee (*Apis mellifera*) gut bacteria across the United States

**DOI:** 10.1128/aem.00755-26

**Published:** 2026-09-21

**Authors:** Casey L. Gregory, Kevin Radja, David C. Haak, Emma L. Bradford, Richard D. Fell, Jenifer B. Walke, Lisa K. Belden

**Affiliations:** 1 Department of Biological Sciences, Virginia Tech 1757, Blacksburg, Virginia, USA; 2 School of Plant and Environmental Sciences, Virginia Tech 1757, Blacksburg, Virginia, USA; 3 Department of Entomology, Virginia Tech 1757, Blacksburg, Virginia, USA; 4 Department of Biology, Eastern Washington University 2380, Cheney, Washington, USA; Norwegian University of Life Sciences, Ås, Norway

**Keywords:** honey bee, antibiotic resistance, tetracycline, ARG, microbiome

## Abstract

Antibiotic use has contributed to antibiotic resistance genes (ARGs) accumulating in many environments, including host-associated microbiomes. Managed honey bee gut bacteria may accumulate ARGs, as honey bees are sometimes treated with antibiotics and often live in agricultural landscapes contaminated with antibiotics. We describe the occurrence and distribution of ARGs in honey bee bacterial gut symbionts from 13 apiaries in a transect across the USA from Washington to Virginia. Using metagenomic sequencing, we detected 55 unique ARGs conferring resistance to 14 classes of antibiotics. Of these, 11 ARGs encoded multidrug resistance. ARGs varied among sites, and ARG composition in hives shifted across the transect. Among honey bee gut bacterial genera, ARG occurrence varied, with *Gilliamella* and *Frischella* containing the highest proportions of ARGs despite their low relative abundance in the gut community, suggesting specific genera may serve as ARG reservoirs. As tetracycline is the most used antibiotic in beekeeping, we compared the frequency and abundance of tetracycline resistance genes across apiaries. All hives contained tetracycline resistance genes, with *tetB* and *tetM* present at all apiaries. Based on qPCR, *tetB* and *tetM* abundance varied significantly among apiaries. *TetB* was higher overall and declined in abundance from Washington to Virginia. We demonstrate that honey bee gut bacteria possess a diversity of ARGs, not all of which are consistent with antibiotic use in beekeeping, ARG frequency varies among bacterial genera in the honey bee gut, and certain ARGs are associated with hive geographic location.

**IMPORTANCE:**

The spread of antibiotic resistance genes (ARGs) to bacterial pathogens is a critical issue facing global health. Gut bacterial symbionts of managed honey bees make good bioindicators for ARGs because honey bees interact with potential environmental reservoirs of ARGs and are broadly distributed across the USA, including in both urban and rural environments. Based on our transect across the USA, ARGs were diverse and widely distributed among honey bee gut symbionts, although certain bacterial genera had a higher propensity for accumulating ARGs. Tetracycline resistance genes were most common, and varied in occurrence and abundance across the transect. The abundance of *tetB*, in particular, increased from east to west along the sampled transect. These large-scale patterns of ARG distribution within a widely dispersed host-associated microbiome system provide a foundation from which to examine the underlying factors driving differences in ARG occurrence and abundance.

## INTRODUCTION

Antibiotics have evolutionary origins in microbial chemical warfare, with some microorganisms producing antimicrobial compounds in response to competition with other microbes for space and resources (1). Approximately 40,000 naturally occurring antibiotics targeting diverse groups of microorganisms have been identified in microbial populations (2). Selection pressure caused by antibiotic production in microbial communities can result in higher survival of microbes with resistance to antibiotics, which is often conferred by antibiotic resistance genes (ARGs). ARGs combat antibiotics via several mechanisms, including modifying target sites to prevent antibiotic binding, expelling antibiotics from the cell interior by way of efflux pumps, reducing membrane permeability to limit antibiotic uptake, and producing enzymes that render antibiotics inactive. ARGs may arise through mutation and be vertically inherited or acquired through horizontal gene transfer. While antibiotics and ARGs naturally occur in microbial populations, antibiotic application by humans in clinical and agricultural settings has contributed to the accumulation of ARGs in a range of systems, including agricultural soils, livestock farms, rivers, coastal waters, wastewater, and human and animal microbiomes (3).

Antibiotics provide a crucial defense against bacterial diseases for humans, livestock, and crops. However, the development and proliferation of ARGs in pathogenic microorganisms is a critical issue threatening global health. Multidrug-resistance (MDR) is of particular concern and has been recognized by the World Health Organization (WHO) as one of the top three major public health threats (2). Identifying trends of ARG occurrence and diversity in systems influenced by anthropogenic activity may help develop strategies for mitigating the spread of ARGs and eventually combating MDR. In this study, we describe ARG occurrence patterns in a widely distributed, semi-domesticated insect pollinator, the European honey bee (*Apis mellifera*).

In the USA, antibiotics are used in managed honey bee colonies to treat outbreaks of the bacterial brood diseases American foulbrood (causative agent: *Paenibacillus larvae*) and European foulbrood (causative agent: *Melissococcus plutonius*) (4). While use in honey bees may not seem like a major input of antibiotics, there are over 2 million managed commercial honey bee hives in the USA alone (5). These bees play a critical role in agriculture, providing essential pollination services to crops like almonds and blueberries, and creating products, such as honey and beeswax. In the USA, the pollination services and products provided by honey bees are valued at over $200 million/year (5). Prior to 2017, antibiotic use for honey bees in the USA was not regulated. Prepared mixtures of tylosin or oxytetracycline were sold to beekeepers, although we do not know how widely these were used. In 2017, the USA FDA issued a mandate that required a prescription from a veterinarian to purchase antibiotics (oxytetracycline, tylosin, or lincomycin) for use in beekeeping (6).

The microbial symbionts associated with honey bees provide important contributions to their health (4). Of particular importance are the bacterial taxa that occupy the guts of honey bees (4). Five taxa, *Snodgrassella, Gilliamella, Bifidobacterium*, *Lactobacillus Firm-4*, and *Lactobacillus Firm-5*, are found in the guts of healthy adult bees and make up the core gut community (4, 7). The gut bacterial communities of honey bees contribute to host health by metabolizing complex macromolecules in the gut, protecting against pathogen invasion, and regulating metabolites in hemolymph (4). Upon emergence from brood cells, adult honey bees lack an established gut bacterial community and acquire gut bacteria through interactions with nestmates and hive material (8). Honey bee gut community structure is associated with age and life stage, indicating that the environment individual bees interact with influences their gut microbiota (9).

The current and past use of antibiotics for the treatment of honey bee diseases has likely contributed to the accumulation of ARGs in honey bee bacterial gut symbionts. For instance, the diversity and prevalence of ARGs in hives in the USA are notably higher than in European hives, where antibiotics are not approved for commercial beekeeping (10). ARGs detected in honey bee gut bacteria are typically associated with the antibiotics most used in beekeeping (11–13), but ARGs associated with resistance to other antibiotics, such as amoxicillin and streptomycin, have also been detected (12, 14–16). This pattern suggests environmental exposures might also result in the occurrence and perpetuation of ARGs in honey bee gut bacteria. Certain agricultural crops pollinated by honey bees, such as apples and pears, are sometimes sprayed with the antibiotics streptomycin and oxytetracycline, which could select for the presence of ARGs (17). The route of gut microbiome acquisition and the lifestyle of honey bees may also contribute to the persistence of ARGs in gut symbionts. Indeed, ARGs conferring resistance to tylosin and lincomycin have been isolated from honey (18), indicating that hive materials can act as ARG reservoirs. Additionally, workers with ARGs in their gut communities may transmit ARGs to other bees through trophallaxis (food exchange) or fecal-oral transmission (8).

Tetracycline is the most common antibiotic used in beekeeping, and tetracycline resistance genes are some of the most frequently identified ARGs in honey bee bacterial symbionts (11, 19–21). The eight tetracycline resistance genes found among honey bee-associated bacteria employ two main mechanisms of resistance: efflux pump genes (*tetB*, *tetC*, *tetD*, *tetH*, *tetL*, *tetY*) remove the drug from the cell, and ribosome protection proteins (*tetM*, *tetW*) alter the antibiotic target (11, 22, 23). Tetracycline resistance genes in honey bee gut bacteria appear to be more prevalent in locations where tetracycline is permitted for beekeeping, although tetracycline resistance genes have been identified in hives from the EU, the Czech Republic, and New Zealand where tetracycline is prohibited (10, 11, 20, 21).

Overall, microbial chemical warfare, historic antibiotic use, and environmental contamination likely act together to perpetuate the existence of ARGs in honey bee-associated gut bacteria. To provide further insight into the repertoire of ARGs present in honey bee gut bacterial symbionts across the USA, we performed metagenomic sequencing on pools of bee guts collected from 89 hives in 13 apiaries across a 3,736 km east-west transect in the USA and analyzed their ARG content. Using these data, we provide an overview of ARG diversity and distribution in honey bee symbionts in the USA. Specifically, we address the following questions: (i) Does ARG presence in honey bee gut bacteria vary across the USA? (ii) Is there variation in ARG occurrence among bacterial members of the honey bee gut microbiome? (iii) Does the abundance of the most commonly identified tetracycline resistance genes vary based on apiary location?

## RESULTS

### Does ARG presence in honey bee gut bacteria vary across the USA?

**Fig 1:**
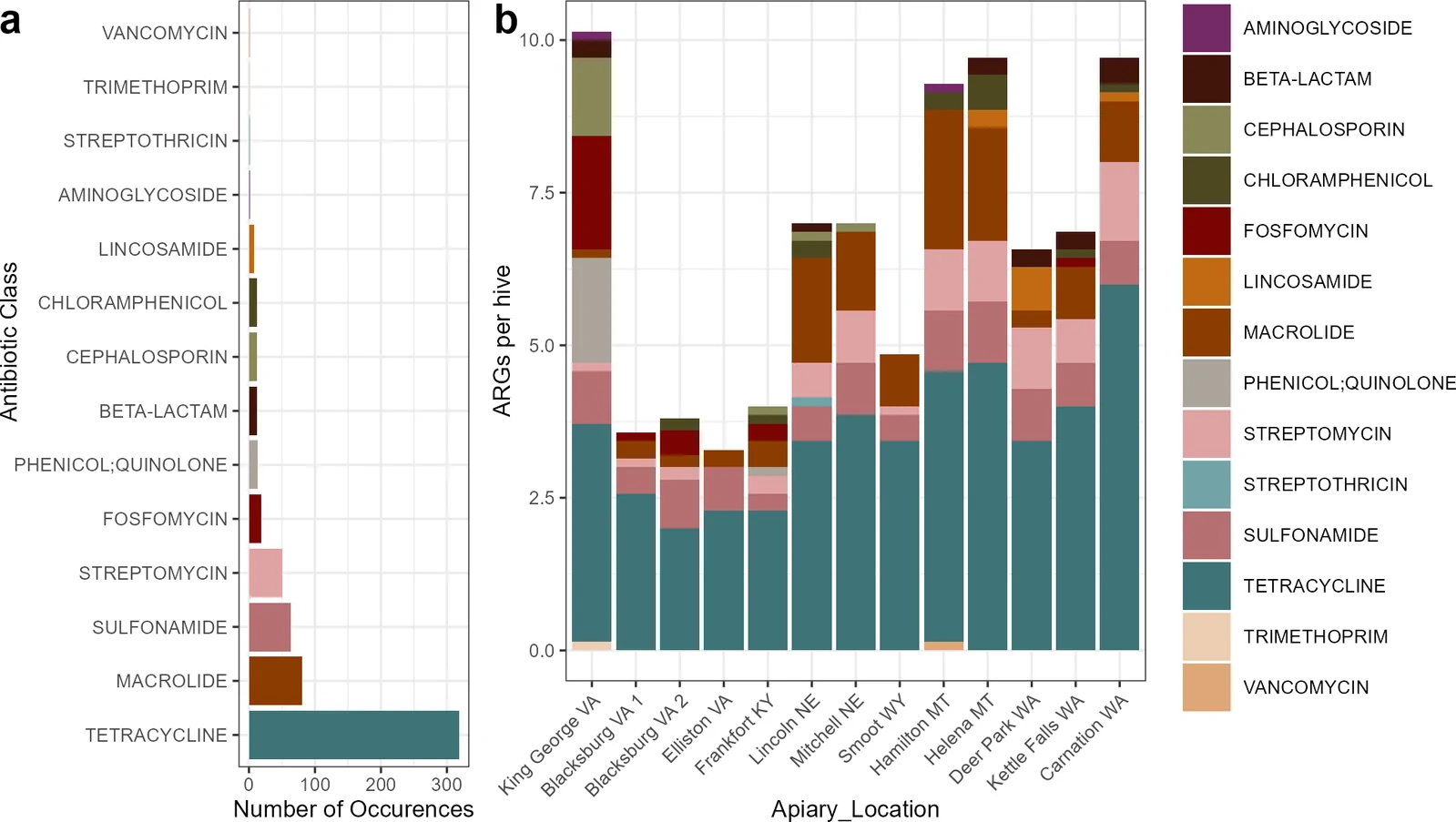
(**a**) Number of ARG hits for each antibiotic class from hives across the USA. (**b**) ARG occurrence per hive by apiary. ARGs are grouped by the class of antibiotic to which the gene confers resistance. ARG count was divided by the total number of sampled hives per apiary to account for sampling depth. Five hives were sampled at the Blacksburg VA 2 location, and seven hives were sampled at all other locations.

**Fig 2:**
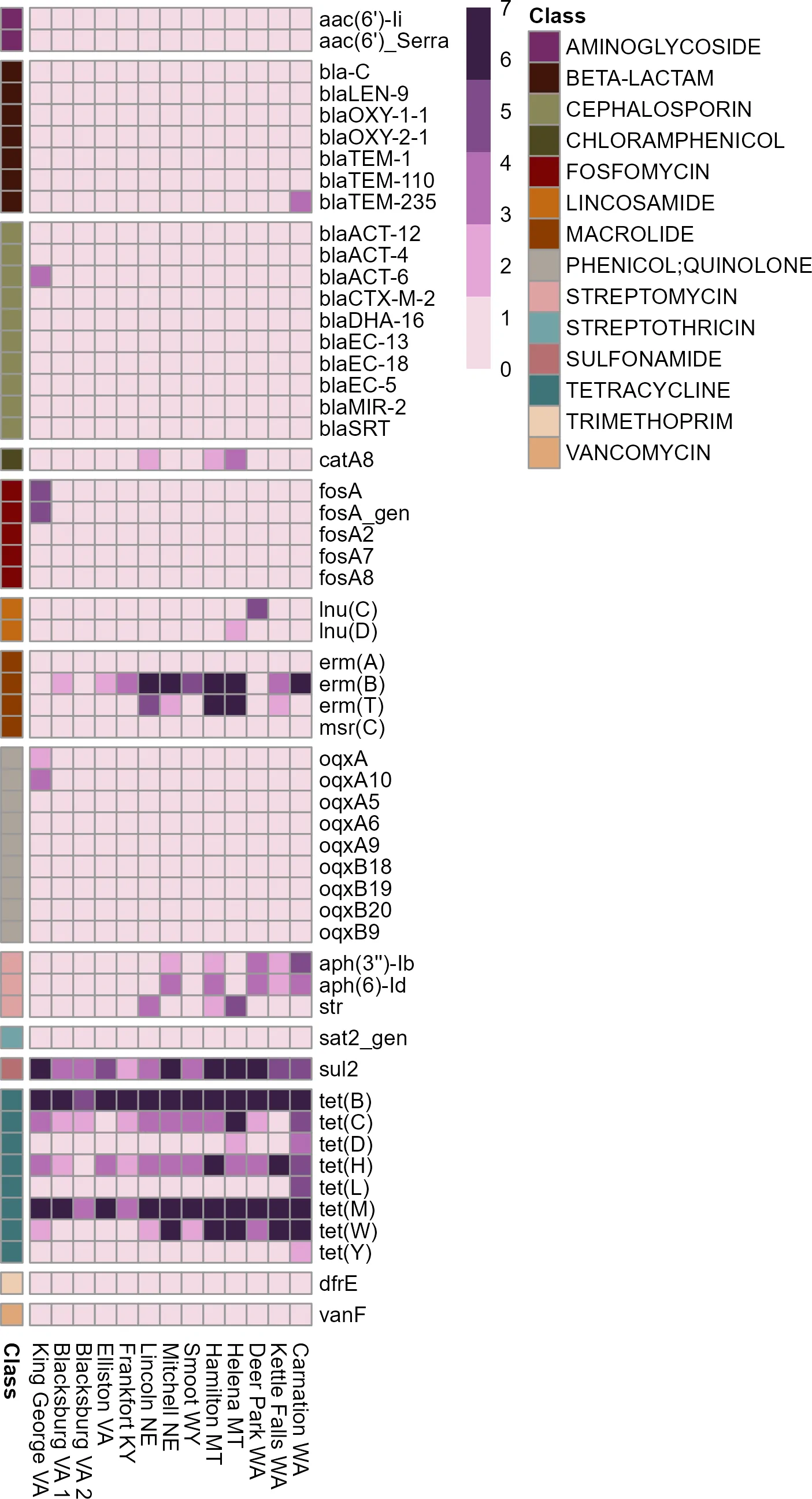
Heatmap showing the number of hives (out of seven for all but Blacksburg VA 2, which was out of five) each ARG occurred in per apiary.

Across all sampled apiaries, we detected a total of 55 unique ARGs that conferred resistance to 14 classes of antibiotics (Fig. 1). Of these, 11 genes were associated with multidrug efflux systems, an indication of multidrug resistance (*oqxA*, *oqxA10*, *oqxA5*, *oqxA6*, *oqxA9*, *oqxB18*, *oqxB19*, *oqxB20*, *oqxB9*, *ermA*, and *ermB*; [24–26]; Fig. 2). At all locations, tetracycline resistance genes were the most common. The antibiotic class with the most diverse ARGs was cephalosporin, with 10 unique genes detected. ARG count (occurrence of unique ARGs/hive) was highest for the apiary located in King George, VA, which contained 71 ARG occurrences across seven hives. Blacksburg VA 2 had the lowest ARG count with 19 occurrences; however, only five hives were sampled at this site.

**Fig 3:**
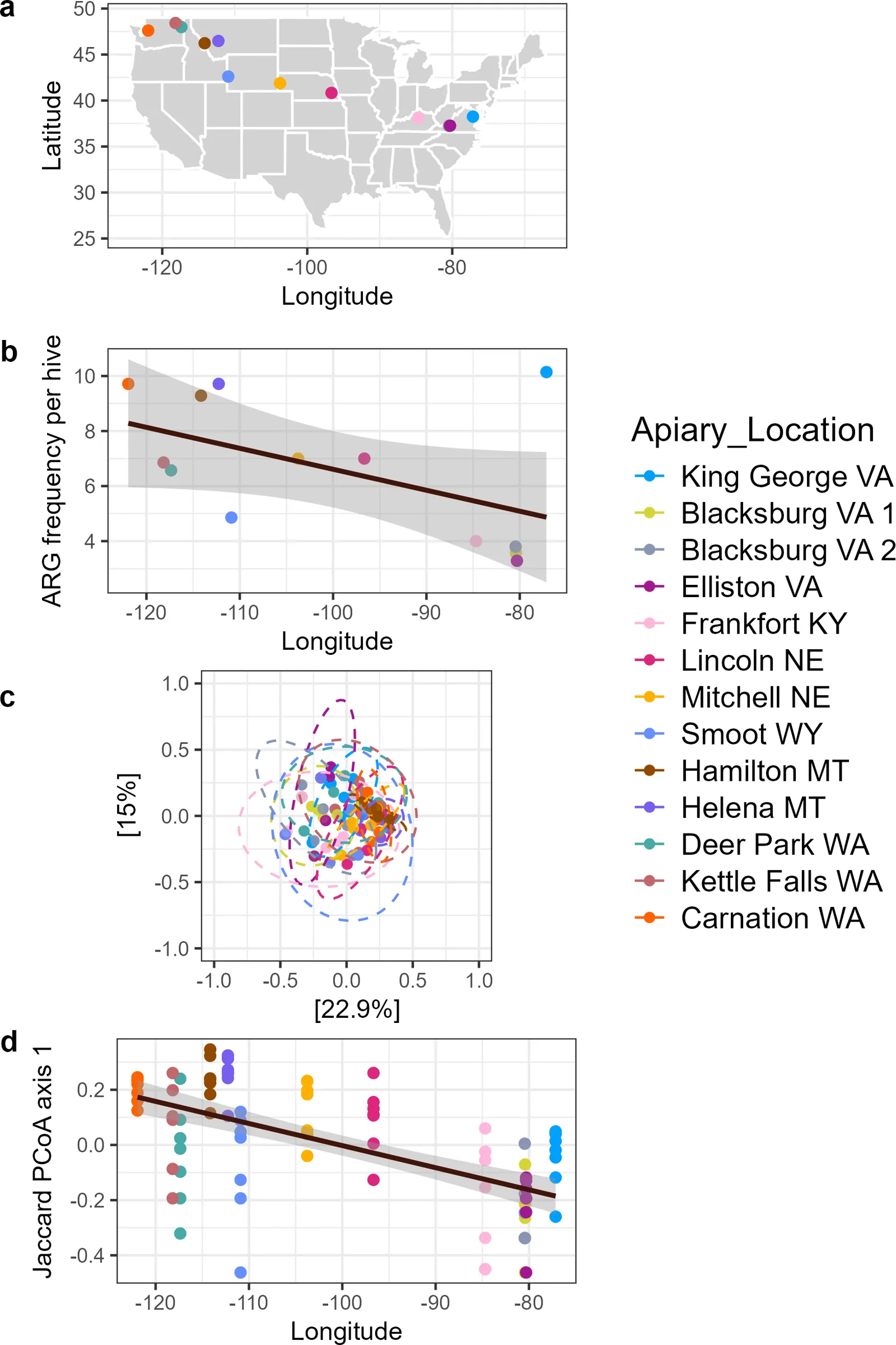
ARG occurrence varied among hives across the USA. (**a**) Map of collection sites. The map was generated using the map_data function in ggplot2. (**b**) Total number of ARG occurrences per apiary was not predicted by longitude. (**c**) PCoA ordination showing Jaccard dissimilarity comparing ARG presence in hives grouped by apiary location. According to Jaccard dissimilarity, ARG composition varied significantly among the sampled apiaries. (**d**) Axis 1 from Jaccard dissimilarity PCoA based on the presence/absence of resistance genes in hives was predicted by longitude.

The total number of ARG occurrences was not significantly correlated with longitude (linear model, beta = −0.08, 95% confidence interval [CI] [−0.16, 8.23e−03], *t* (11)= −1.99, *P* = 0.072; Std. beta = −0.51, 95% CI [−1.08, 0.06]; Fig. 3b). The composition of ARGs present varied by apiary according to Jaccard dissimilarity despite considerable overlap in composition across sites (permutational multivariate analysis of variance [PERMANOVA] by site, df = 12, SumofSqs = 6.2525, *R*^2^ = 0.34589, F = 3.3491, *P* = 0.001; Fig. 3c). Jaccard dissimilarity was also positively correlated with apiary distance, meaning that apiaries that were closer together tended to be more similar in ARG composition, although the effect was relatively small (Mantel test, *R* = 0.0763, *P* = 9e−04). The Jaccard PCoA axis one was negatively correlated with longitude (linear model, beta = −8.11e−03, 95% CI [−0.01, −5.85e−03], *t* (87) = −7.05, *P* < 0.001; Fig. 3d), indicating a general shift in ARG composition across the USA.

### Is there variation in ARG occurrence among members of the honey bee gut microbiome?

**Fig 4:**
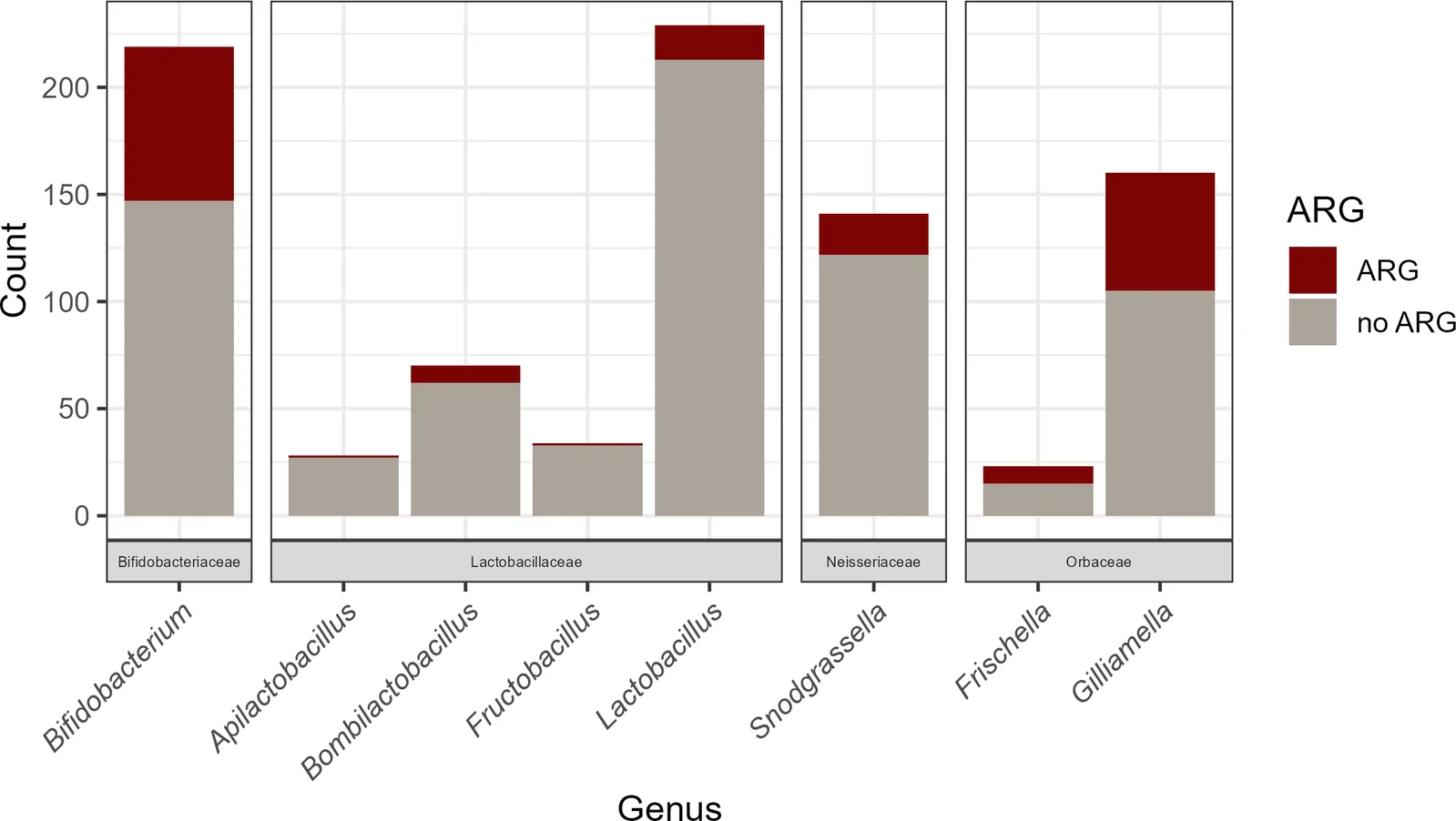
ARGs detected in honey bee gut bacterial genera. Taxonomic bins assigned to honey bee gut genera across all sampled apiaries. Bins containing ARGs are shown in red.

Of the eight examined honey bee-associated bacterial genera, *Frischella* and *Gilliamella* contained the highest proportion of taxonomic bins containing ARGs, with 34.8 and 34.4%, respectively. *Bifidobacterium* also had a relatively large proportion of ARGs at 32.9%. *Apilactobacillus* and *Fructobacillus* contained the lowest proportions, with 3.6 and 3.0%, respectively. These ARG proportions significantly varied by genus (linear mixed model, 95% CI [0.07, 0.13], *t* (135) = 6.89, *P* < 0.001; Fig. 4). ARG proportion did not appear to scale with overall relative abundance of the genera in the data set, as *Frischella* and *Gilliamella* did not have the highest relative abundance. *Lactobacillus*, *Bombilactobacillus*, and *Snodgrassella* contained relatively moderate proportions of ARGs at 6.97, 11.4, and 13.5%, respectively.

### Does the abundance of the most commonly identified tetracycline resistance genes vary based on apiary location?

**Fig 5:**
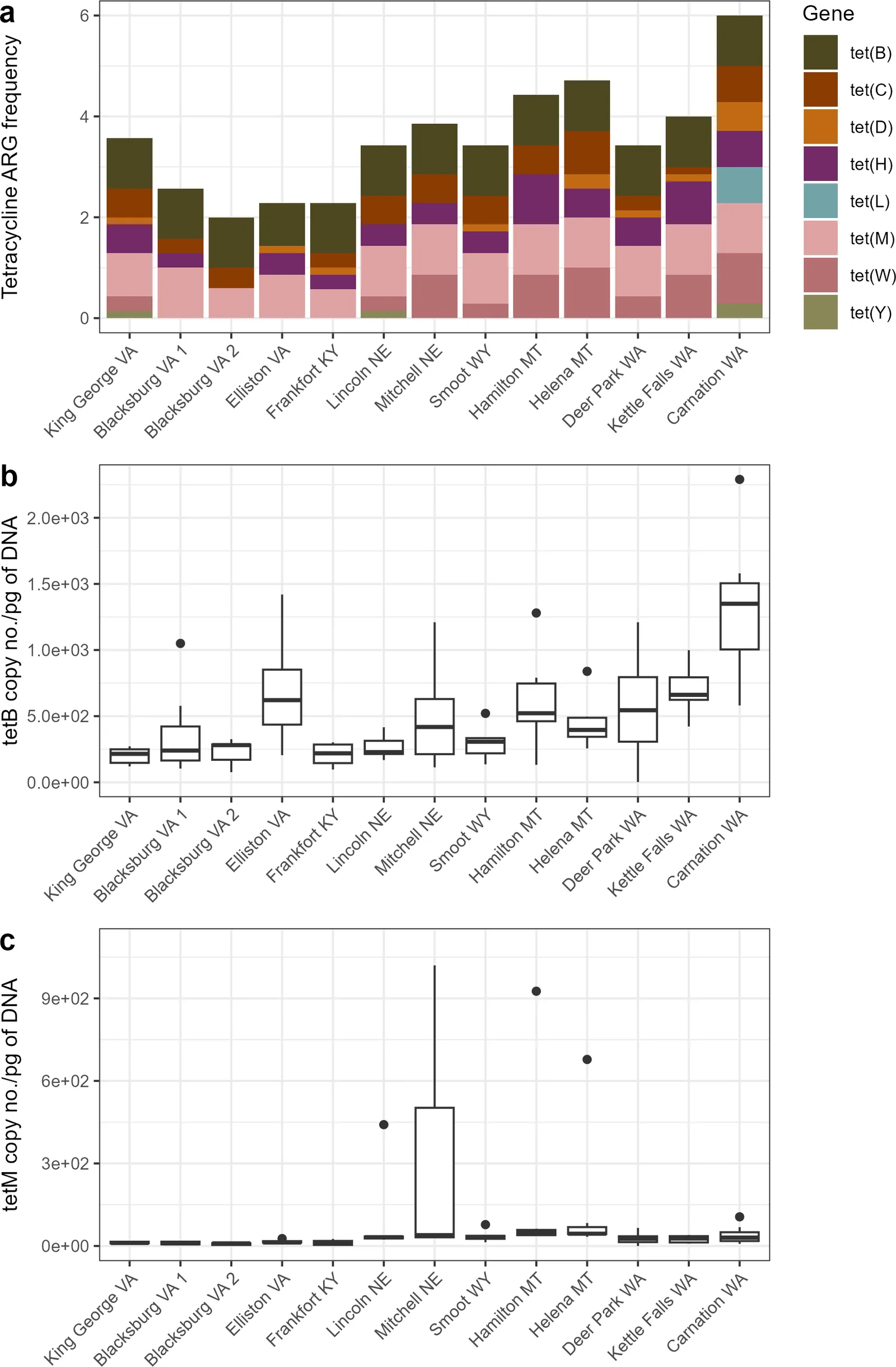
(**a**) Tetracycline resistance gene occurrence per hive across the USA. Occurrence of the eight detected tetracycline resistance genes at each apiary was divided by the number of hives sampled at each respective apiary, giving tetracycline resistance gene frequency per hive. (**b**) Total copy number of *tetB* determined using qPCR and standardized based on total DNA concentration in each sampled apiary. Boxes represent median with 1st and 3rd quartiles. (**c**) Total copy number of *tetM* determined using qPCR and standardized based on the total DNA concentration across the apiary. One hive from Deer Park, WA, that was an extreme outlier (2,730 copies/pg DNA) was removed for visual clarity. Boxes represent median with 1st and 3rd quartiles.

**Fig 6:**
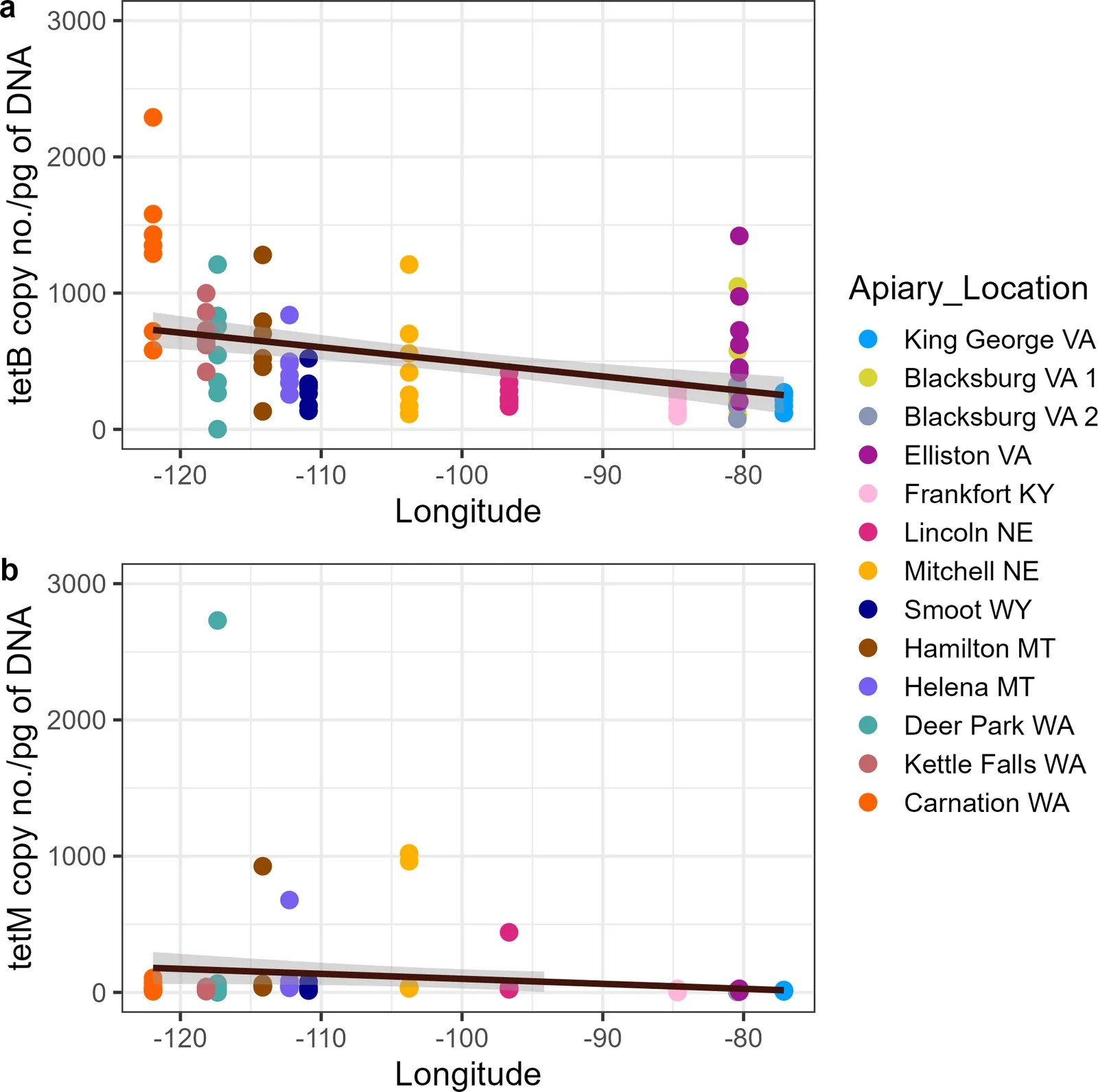
Spatial trends of *tetB* and *tetM* abundance in honey bee hives across a USA west-east transect. (**a**) Standardized *tetB* copy number was negatively correlated with longitude. (**b**) Standardized *tetM* copy number was not predicted by longitude.

Tetracycline is the most common class of antibiotics used in beekeeping, so we analyzed the diversity of the tetracycline resistance genes in our data set across apiaries. We identified eight unique tetracycline resistance genes in our sampled hives: *tetB*, *tetC*, *tetD*, *tetH*, *tetL*, *tetM*, *tetW*, and *tetY* (Fig. 5a). The apiary in Carnation, WA, contained the highest frequency of tetracycline resistance genes and had the most tetracycline resistance gene diversity. Indeed, Carnation, WA, was the only apiary to have all eight tetracycline resistance genes present. *TetB* and *tetM* were present in all apiaries. Since *tetB* and *tetM* were present at all sampled sites, we compared their copy numbers across apiaries using qPCR. Our reaction efficiencies for these assays were relatively low (62–87% for *tetB* assay and 47–92% for *tetM* assay), so we used these results only to compare relative values among our apiaries and not to speculate on the absolute copy numbers relative to other studies. Both *tetB* and *tetM* copy numbers varied significantly among apiaries, but *tetB* was more abundant overall (*tetB*, Welch’s one-way ANOVA, *F* = 6.8116, num df = 12, denom df = 28.937, *P* = 1.226e–05 | *tetM* Welch’s one-way ANOVA, *F* = 2.2249, num df = 12, denom df = 28.945, *P* = 0.03871 | Fig. 5b and c). Since sites were sampled on a west-east transect, we also examined whether *tetB* and *tetM* abundance correlated with longitude. *TetB* abundance was negatively correlated with longitude, with *tetB* abundance higher in the western apiaries (*tetB*, linear mixed model, beta = −10.69, 95% CI [−15.46, −5.92], *t* (87) = −4.45, *P* < 0.001; Std. beta = −0.43, 95% CI [−0.62, −0.24] | Fig. 6a). *TetM* abundance was not significantly correlated with longitude (*tetM*, linear mixed model, beta = −3.66, 95% CI [−7.98, 0.66], *t* (87) = −1.68, *P* = 0.096; Std. beta = −0.18, 95% CI [−0.39, 0.03] | Fig. 6b).

## DISCUSSION

### Honey bee gut bacterial communities possess a diverse array of ARGs, not all of which are consistent with antibiotic practices in beekeeping

We detected a wide variety of ARGs within gut bacterial communities of managed honey bees from across the USA. Many of these have been documented in honey bees, including those providing resistance to tetracycline, lincomycin, macrolides, streptomycin, and vancomycin (11, 14, 19, 20, 22). Across the transect, there was considerable overlap in ARGs present in the sampled hives. However, ARG composition varied among apiaries, and we observed spatial trends in ARG composition. Apiaries in closer proximity to one another had more similar ARG profiles, and ARG composition was associated with longitude. Similar trends have been reported in apiaries across China and Italy, with notable overlap in ARG composition accompanied by disparity in the geographic distribution of certain ARGs (19, 20, 27). The ARG profiles detected in the gut microbiomes of honey bees in our transect were similar to those reported for environmental sources, including farms, wastewater treatment plants, water, and soil (3), as well as broadly mirroring previously reported profiles from livestock, such as swine, cattle, and poultry (3, 28). For instance, tetracycline, macrolide, sulfonamide, and streptomycin ARGs are prevalent in agricultural settings and in the environment (3, 28) and also common in honey bee gut microbiomes. Our findings appear to corroborate other studies demonstrating that honey bees have disproportionately greater ARG abundance and diversity in their symbiotic bacterial communities than other social bees, such as *Apis cerana* and *Bombus terrestris* (27), highlighting how anthropogenic influence may shape antibiotic resistance in host-associated microbial communities.

We identified 11 genes associated with multidrug resistance. Nine of these encode *oqxAB* efflux pumps, which provide resistance to fluoroquinolones, as well as cephalosporin and aminoglycoside (24, 29). The other two multidrug resistance genes, *ermA* and *ermB*, are erythromycin resistance genes that provide resistance to macrolide-lincosamide-streptogramin antibiotics (25, 26). The occurrence of multidrug resistance genes in honey bee gut symbionts is concerning since humans interact closely with managed honey bee colonies. There is some evidence that interactions with livestock can influence human gut microbiota structure and antibiotic resistance (30). In a broader context, the acquisition and spread of multidrug resistance genes in honey bee gut bacteria across the USA may be reflective of increased environmental spread of multidrug resistant bacteria, which represent a major health risk if they are passed to potential human pathogens.

The hives in our study were reported to have not been treated with antibiotics at least 6 months prior to sampling. Regardless, the highest frequency of ARGs in our data set was from tetracycline and macrolide, which represent the most used antibiotics (oxytetracycline and tylosin, respectively) in beekeeping in the USA. Although antibiotics were not recently applied to the sampled hives, historic beekeeping practices may have contributed to the presence of ARGs. Antibiotic resistance can persist long after exposure and can even occur in hives never treated directly with antibiotics (10, 11, 15, 31). Treatment of nearby hives could have contributed in part to ARG accumulation. Honey bees sometimes rob honey and nectar from weak colonies in close proximity to their own hive (32). Robbers may transmit antibiotic residues or antibiotic-resistant bacteria between hives and perpetuate ARGs in honey bee bacteria. Additionally, queens sourced from different breeders have differing gut microbiomes (33), and those sourced from colonies with historic antibiotic use may pass ARGs to other members of the colony.

Another potential route of ARG acquisition for honey bees is interaction with environmental reservoirs of ARGs, such as when drinking or foraging. Honey bees prefer water from stagnant pools containing dissolved salts, and, thus, will collect water from sewage effluent and other wastewater sources that may also harbor an abundance of ARGs (34). Interestingly, Fry et al. (35) found Class 1 integron prevalence, a universal indicator of antimicrobial resistance, was correlated with waterbody area in the honey bee foraging radius. Foraging may also expose honey bees to ARGs that are not typically used in beekeeping. Streptomycin ARGs were found at all but one of our apiaries. In the USA, streptomycin is used to treat bacterial diseases that impact crop production, such as fireblight in apples and pears, and spraying typically occurs during bloom when bees are actively foraging (36). It is possible foragers acquire streptomycin resistance through direct contact or interactions with streptomycin resistant bacteria on agricultural crops. Indeed, honey bees have hairs on their body that aid in pollen collection and can accumulate other small particles, including microorganisms (22, 37).

While antimicrobial resistance is typically discussed in the context of selection resulting from antibiotic use, it is possible for resistance to occur spontaneously. Antimicrobial resistance initially evolved in the absence of human-produced antibiotic drugs, and selection pressures other than antibiotic exposure may influence resistance patterns (38, 39). For example, Knöppel et al. (39) demonstrated that selection based on growth media influenced antibiotic resistance, despite the growth media containing no antibiotics. Therefore, environmental conditions not related to direct antibiotic application may drive ARG accumulation in some bacterial communities. In addition, interspecies interactions in microbial communities can impact resistance (40). Some bacteria can reduce the concentration of antibiotics in the environment or alter the state (e.g., decrease the metabolic rate) of surrounding cells, which increases community stability in the presence of antibiotics (40). Thus, ARG accumulation in host-associated microbial communities, including the honey bee gut microbiome, may also be influenced by environmental conditions unrelated to antibiotic exposure and interspecies interactions.

### ARG occurrence differed among members of the honey bee gut microbiome and may be related to taxonomy

ARG occurrence varied among members of the honey bee gut microbiome. Among the genera we tested, *Gilliamella* and *Frischella* contained the highest proportions of ARGs at 34% each. *Bifidobacterium* also had a relatively high proportion at 32%. In a similar study conducted on apiaries from China, Sun et al. (6) also found that *Gilliamella* and *Bifidobacterium* possessed high levels of ARGs. A possible explanation for the large proportion of ARGs in *Gilliamella* and *Frischella* could be their location within the honey bee gut. *Gilliamella* and *Frischella* occupy the pylorus/ileum of honey bees rather than the rectum where the majority of bacteria are found (4). The location of *Gilliamella* and *Frischella* may result in exposure to higher or more potent doses of ingested antibiotics, which could lead to selection and ultimate accumulation of ARGs. However, *Snodgrassella* resides in the ileum alongside *Gilliamella* and *Frischella*, but had a relatively moderate proportion of ARGs at 13.5%. In addition, *Bifidobacterium* had a proportion of ARGs closer to those of *Gilliamella* and *Frischella,* but resides in the rectum. Given this, a more likely explanation is that evolutionary history influences ARG accumulation and perpetuation.

Different bacterial genera could have a higher propensity to accumulate ARGs. Indeed, the frequency of spontaneous mutations that provide resistance to antimicrobials varies based on bacterial species (38). *Frischella perrara* has exhibited resistance to ampicillin, carbenicillin, streptomycin, and oxytetracycline in disc diffusion assays (41), which suggests the genus can accumulate a variety of ARGs. There is evidence that closely related bacteria more readily engage in horizontal gene transfer, which could facilitate the accumulation of ARGs in related taxa (42). G*illiamella,* in particular, has been shown to engage in horizontal gene transfer of a transposon associated with tetracycline resistance (10). *Gilliamella* and *Frischella* are both members of the family *Orbaceae*. The similarity in ARG proportion between these genera could suggest a genetic predisposition to harbor ARGs. Genera within the family *Lactobacillaceae* also contained relatively similar proportions of ARGs, which supports the idea that shared evolutionary history may impact the propensity to accumulate ARGs. *Bifidobacterium* was the only genus we analyzed from the family *Bifidobacteriaceae*; therefore, we could not compare whether genera in this family followed the same trend. Evolutionary history playing a role in ARG distribution has been demonstrated in other systems, such as aquaculture ponds and compost (43, 44). In these systems, ARG diversity and abundance correlate with the taxonomic composition of bacterial communities (43, 44). Therefore, our results support the growing body of evidence that suggests phylogeny influences ARG accumulation in microbial communities.

### Tetracycline resistance genes are most common among honey bee gut symbionts, and the abundances of *tetB* and *tetM* vary across the USA

ARGs that provide resistance to tetracycline class antibiotics were also some of the most diverse, with eight unique genes identified. The eight tetracycline resistance genes detected in this study were also found previously in USA honey bee colonies (11). Tian et al. (11) also detected tetracycline resistance genes in managed hives from WA (overlapping with our study), as well as CT, MD, AZ, and FL, and found that there was variation among hives. Our findings added to those of Tian et al. (10) suggest tetracycline resistance genes have persisted in populations of managed honey bees in the USA for over ten years. Moreover, with the exception of WA, our study covered hives from different regions, emphasizing that tetracycline resistance genes are widely distributed in hives throughout the USA.

Of the eight tetracycline resistance genes we detected, *tetB* and *tetM* were the only tetracycline resistance genes found in every apiary. Both *tetB* and *tetM* abundance differed across sites. However, *tetB* abundance was generally higher and more variable. *TetB* and *tetM* utilize different mechanisms of resistance, with *tetB* encoding an efflux pump protein and *tetM* encoding a ribosomal protection protein (23). Efflux pump proteins often occur on mobile genetic elements, such as plasmids and transposons (45). Transposable elements are readily transferred among bacteria and often drive the adaptation and evolution of bacterial communities (46). Therefore, variability and higher abundance of *tetB* among apiaries may reflect how transposable elements aid in bacterial community adaptation.

### Conclusions

Antibiotic resistance threatens human health when human pathogens become resistant to antibiotics. Monitoring the dissemination of ARGs and resistant bacteria in the environment is important to the control of antibiotic resistance. The interface between the environment and host-associated microbial communities represents a critical junction for the emergence, persistence, and spread of antibiotic resistance (47). The presence of ARGs in honey bee bacterial symbionts indicates that managed honey bee colonies could be used for monitoring ARG distribution in the environment, and, indeed, numerous studies have suggested honey bees for this type of biomonitoring (14, 15, 19, 20, 22, 27, 35, 48, 49). Honey bees are a good choice for bioindication of ARGs because they are easily managed by humans, are already present in managed colonies across the entire country, and thrive in a variety of environments. Honey bees are also central place foragers (50), collecting resources from the environment within an approximate 3 km radius around the hive, and recent modeling has suggested that there are correlations between antibiotic resistance profiles in honey bee bacteria and land use (51). Honey bees, therefore, could already be used for monitoring ARG development and spread across the USA by sampling hives placed near potential environmental sources of ARGs.

We demonstrate several trends of ARG distribution within a widely dispersed host-associated microbiome system. Specifically, we show that different bacterial taxa, regardless of their abundance in a community, seem to vary in their propensity to harbor ARGs. Such patterns could be a product of ecological niche (e.g., location in the gut influences antibiotic exposure) or some other underlying genetic mechanism. Further research is needed to understand the factors that influence ARG accumulation and perpetuation in different bacterial taxa. We also show that ARGs that provide resistance to the same class of antibiotic can vary in distribution and abundance. Variability among ARG abundance and presence within a single antibiotic class could be caused by how the genes are transferred among microbial community members (e.g., plasmids, transposable elements). Our study describes wide-scale patterns of ARG distribution across the USA, thus expanding our understanding of ARG spread and persistence in microbial populations and sets the stage for subsequent investigations into antimicrobial resistance in animal-associated bacterial symbionts.

## MATERIALS AND METHODS

### Honey bee collection

Honey bees were collected from 13 sites across the USA (Fig. 3a) in June 2022, some by us directly and others by beekeepers who contributed bees to this project. Specific sites were selected based on research contacts and by contacting beekeeping groups across the USA. For every site, a survey was completed, and a photograph of the apiary was taken. The survey was used to collect standardized data about each site and the hives sampled. As part of the survey, we asked whether the sampled hives had been treated with antibiotics since the start of the year (January 2022). None of the beekeepers reported antibiotic use during 2022. From every site, except Blacksburg VA 2, seven healthy overwintered hives were sampled. Five hives were sampled at the Blacksburg VA 2 site. From each hive (total = 89 hives), we obtained two 50 mL conical sterile centrifuge tubes filled with adult bees from a frame just outside the brood nest. Following collection, they were kept on ice in the field and either returned to our lab (for samples we collected locally) or were frozen until shipping. Samples were shipped on dry ice to Virginia Tech (Blacksburg, VA, USA). Once samples arrived at Virginia Tech, they were transferred to a −70°C freezer until sample processing.

### DNA extraction

DNA extraction was performed at the hive level, with five random honey bee workers per hive pooled. Individual workers were briefly thawed and surfaced sterilized in 5% bleach, followed by three rinses in sterile Ultrapure water, and whole guts (midgut to hindgut) dissected (52). Dissected whole guts were placed in 180 µL DNA lysis buffer (consisting of 20 mM Tris-HCl pH 8, 2 mM EDTA pH 8, and 1.2% Triton-X-100) with lysozyme (20 mg lysozyme per 1 mL lysis buffer) in pools of five honey bee guts per hive, homogenized using sterile plastic pestles, and incubated at 37°C for 1 h (51). DNA extraction was done following the Qiagen DNeasy Kit protocol (Qiagen, Germantown, MD, USA) using 200 µL of molecular-grade water for the final DNA elution. DNA was quantified using a Qubit 2.0 fluorometer (Invitrogen, Carlsbad, CA, USA), and a standardized volume of 50 µL per hive was sent for metagenomic sequencing at the Duke Center for Genomic and Computational Biology (Duke University, Durham, NC, USA).

### Metagenome sequencing, assembly, and binning

High-throughput sequencing of DNA from extracted bee guts was performed on the NovaSeq 6000 platform via two 2 × 150 bp S-Prime flow cells, generating over 600 Gbp each. Samples ranged from 3.4 to 8.7 Gbp with a mean of 6.1 Gbp and an average Q30 of 87%. Raw paired-end sequences were merged across lanes into forward and reverse reads for each sample. To remove host contamination, these reads were aligned to the honey bee (*Apis mellifera*) reference genome Amel_4.5 from National Center for Biotechnology Information (NCBI) RefSeq (https://www.ncbi.nlm.nih.gov/datasets/genome/GCF_000002195.4/) using the Burrows-Wheeler alignment tool BWA-MEM (53, 54). The reads mapped to Amel_4.5 were filtered with SAMtools to obtain non-bee reads (55). For the remaining non-host reads, we employed the SqueezeMeta (56) pipeline for metagenome processing, including assembly and binning. SqueezeMeta was run sequentially, treating all samples individually with the rest of the parameters set to their default. The default assembler for SqueezeMeta, Megahit, was selected due to its performance with Illumina short reads. Assembly resulted in an average N50 of 1,765 bp with an average total length of 50 Mbp.

### *TetB* and *tetM* qPCR

As *tetB* and *tetM* were the most commonly occurring tetracycline resistance genes across our sampled sites, we performed qPCR assays to determine their abundance in the sampled hives. Primers used for *tetM* and *tetB* were developed by Aminov et al. (57–59) and previously used in honey bee-associated bacteria by Tian et al. (11) (Table 1). We developed gBlock gene fragments (Integrated DNA Technologies, Coralville, Iowa, USA) to use as standards in each of the two SYBR assays (Table 1). For each run, a standard curve was made based on 10-fold dilutions (10^9^ to 10^3^ gene copies) of the appropriate gBlock gene fragment (Integrated DNA Technologies, Coralville, Iowa, USA). The total volume of each reaction was 15 μL, containing 3 μL molecular water, 7.5 μL SsoAdvanced Universal SYBR Green Supermix (Bio-Rad Labs, Hercules, CA, USA), 0.75 μL of each primer (10 μM), and 3 μL template DNA or gBlock standard. Samples were run in singleton per hive, while standards and no-template controls were run in triplicate. A CFX96 Touch Real-Time PCR Detection System (Bio-Rad Labs, Hercules, CA, USA) was used for each reaction. The *tetB* reaction used the conditions: 94°C for 1 min, followed by 45 cycles of 94°C for 30 s, 65°C for 1 min, and 72°C for 1 min (57, 58). The *tetM* reaction used the conditions: 94°C for 10 min, followed by 45 cycles of 94°C for 15 s, 55°C for 1 min, and 61°C for 1 min (59). The tetB assay had a distinct melt curve at 84°C (SD = 0.62). We did not include replicates that had a melt curve value less than 83°C or greater than 85°C. The tetM assay had an average melt curve temperature of 81.5°C (SD = 1.52), and we excluded values lower than 80°C and higher than 85°C. Our reaction efficiencies were 62–87% for the tetB assay and 47–92% for the tetM assay. The number of gene copies in each sample was calculated based on the run’s standard curve. *TetB* and *tetM* copy number were standardized by dividing the gene copy number by DNA concentration (pg/μL) in each 3 μL sample.

**TABLE 1:** Sequences of the primers and gBlock standards used for the *tetB* and *tetM* qPCR assays

	Forward primer	Reverse primer	Reference	gBlock standard
*TetB*	TACGTGAATTTA TTGCTTCGG	ATACAGCATC CAAAGCGCAC	Aminov et al. (58), Aminov et al. (57)	CCCAGTACGTGAATTTATTGCTTCGGTAGGGAATCTTCCGCAATGGACGAAA GTCTGACGGAGCAACGCCGCGTGAGTGATGAAGGTTTTCGGATCGTAAAG CTCTGTTGTTAGGGAAGAACAAGTGCAAGAGTAACTGCTTGCACCTTGACG GTACCTAACCAGAAAGCCACGGCTAACTACGTG ATACAGCATCCAAAGCGCACACGTAGG
*TetM*	ACAGAAAGC TTATTATATAAC	TGGCGTGTC TATGATGTTCAC	Aminov et al. (59)	CCCAGACAGAAAGCTTATTATATAACTAGGGAATCTTCCGCAATGGACGAAA GTCTGACGGAGCAACGCCGCGTGAGTGATGAAGGTTTTCGGATCGTAAAGCTCT GTTGTTAGGGAAGAACAAGTGCAAGAGTAACTGCTTGCACCTTGACGGTACCTAA CCAGAAAGCCACGGCTAACTACGTGTGGCGTGTCTATGATGTTCACACGTAGG

### ARG analyses

#### Does ARG presence in honey bee gut bacteria vary across the USA?

Antibiotic resistance genes were identified with ABRicate v1.0.0 (60) using standard parameters. ABRicate determines ARG presence within a sample. Since each metagenome sample represented an individual hive, ARG occurrence (presence/absence of each of the identified ARGs in the data set) was counted at the hive level and compared across apiaries. The number of occurrences of each ARG was tallied per apiary and divided by the total number of sampled hives in the apiary (seven hives for all apiaries, except Blacksburg VA 2, which had five hives) to give us ARG frequency per hive. In addition, the total number of ARG occurrences was divided by the number of sampled hives. All statistical analyses were completed in R (v4.5.0 [61]). A linear model (estimated using OLS) was performed to determine whether longitude predicted ARG frequency per hive. To further evaluate differences in ARG occurrence across the sampled apiaries, a hive by ARG presence/absence matrix was assembled. Jaccard dissimilarity was calculated using Phyloseq (v.1.38.0) (62) and visualized on a PCoA ordination. A PERMANOVA (function adonis2; vegan package v2.5-7 [63]) based on 999 permutations was then run to compare ARG composition across apiaries. To determine whether apiaries in closer proximity had greater similarities in ARG composition, we performed a Mantel test (function mantel; vegan package v2.5-7 [63]) on the Jaccard dissimilarity matrix and the Haversine distances of the apiaries (function distm; geosphere package v1.6-8 [64]).

#### Is there variation in ARG occurrence among members of the honey bee gut microbiome?

To determine whether ARG occurrence varied among bacterial taxa in the honey bee gut microbiome, we examined the proportion of taxonomic bins assigned to honey bee gut members that contained ARGs. We focused this analysis on bacterial genera associated with the honey bee gut microbiome that were highly abundant in our data set. We selected the core genera of the honey bee gut microbiome: *Bifidobacterium*, *Gilliamella*, *Lactobacillus*, and *Snodgrassella* (4, 7). We also included non-core honey bee-associated genera in the same families as at least one of the core members: *Apilactobacillus*, *Fructobacillus*, and *Bombilactobacillus* from the family *Lactobacillaceae* and *Frischella* from the family *Orbaceae*.

For the selected genera, contigs labeled as ARGs by ABRicate (60) were selected from the taxonomic bins created using SqueezeMeta (56). The raw reads were then aligned to the indexed ARG contigs using BWA-MEM (54, 65), and the read counts were generated using SAMtools (55). The total number of bins assigned to a particular bacterial genus was summed across all apiaries, and then for each genus, the number of bins containing at least one ARG was counted. The proportion of ARG-containing bins for each genus was then calculated by dividing the number of ARG-containing bins by the total number of bins assigned to each genus. To determine whether there was significant variability in ARG occurrence across the selected genera, we fit a linear mixed model (estimated using REML and nloptwrap optimizer) to predict ARG proportion by genus. The model included apiary as a random effect. Standardized parameters were obtained by fitting the model on a standardized version of the data set. Accordingly, 95% confidence intervals (CIs) and *P*-values were computed using a Wald *t*-distribution approximation.

#### Does the abundance of the most commonly identified tetracycline resistance genes vary based on apiary location?

Since tetracycline resistance genes were the most common across all sites, we analyzed the diversity and occurrence of tetracycline resistance genes among the sampled apiaries. We first partitioned the ABRicate output to include only the eight genes detected in our data set that provide resistance to tetracycline. As with the overall ARG data, we summed each tetracycline gene count by apiary and divided the count by the number of hives sampled in that apiary. The most commonly occurring tetracycline resistance genes, *tetB* and *tetM*, were quantified in a qPCR assay. Standardized gene copy number (copy number/picogram of DNA) for *tetB* and *tetM* were compared across apiaries using one-way Welch’s ANOVAs. Additionally, we fitted linear models (estimated using OLS) to predict copy number of *tetB* and *tetM* per picogram of DNA with longitude.

## Data Availability

Data underlying this manuscript are accessible through the Virginia Tech Data Repository at https://doi.org/10.7294/28774307 and deposited with NCBI under PRJNA1510816.

## References

[1] Cornforth DM, Foster KR. 2015. Antibiotics and the art of bacterial war. Proc Natl Acad Sci USA 112:10827–10828. doi:10.1073/pnas.1513608112 PMC4568221 26305963

[2] Gajic I, Kekic D, Jankovic M, Tomic N, Skoric M, Petrovic M, Mitic Culafic D, Opavski N, Ristivojevic P, Krstic Ristivojevic M, Lukovic B. 2025. Nature’s arsenal: uncovering antibacterial agents against antimicrobial resistance. Antibiotics (Basel) 14:253. doi:10.3390/antibiotics14030253 PMC11939603 40149065

[3] Zhuang M, Achmon Y, Cao Y, Liang X, Chen L, Wang H, Siame BA, Leung KY. 2021. Distribution of antibiotic resistance genes in the environment. Environ Pollut 285:117402. 10.1016/j.envpol.2021.117402. 34051569

[4] Raymann K, Moran NA. 2018. The role of the gut microbiome in health and disease of adult honey bee workers. Curr Opin Insect Sci 26:97–104. doi:10.1016/j.cois.2018.02.012 PMC6010230 29764668

[5] National Agricultural Statistics Service. 2025. Honey. United States Department of Agriculture

[6] Richards ED, Tell LA, Davis JL, Baynes RE, Lin Z, Maunsell FP, Riviere JE, Jaberi-Douraki M, Martin KL, Davidson G. 2021. Honey bee medicine for veterinarians and guidance for avoiding violative chemical residues in honey. J Am Vet Med Assoc 259:860–873. doi:10.2460/javma.259.8.860 34609191

[7] Zheng J, Wittouck S, Salvetti E, Franz C, Harris HMB, Mattarelli P, O’Toole PW, Pot B, Vandamme P, Walter J, Watanabe K, Wuyts S, Felis GE, Gänzle MG, Lebeer S. 2020. A taxonomic note on the genus *Lactobacillus*: description of 23 novel genera, emended description of the genus *Lactobacillus* Beijerinck 1901, and union of lactobacillaceae and leuconostocaceae. Int J Syst Evol Microbiol 70:2782–2858. doi:10.1099/ijsem.0.004107 32293557

[8] Powell JE, Martinson VG, Urban-Mead K, Moran NA. 2014. Routes of acquisition of the gut microbiota of the honey bee apis mellifera. Appl Environ Microbiol 80:7378–7387. doi:10.1128/AEM.01861-14 PMC4249178 25239900

[9] Hroncova Z, Havlik J, Killer J, Doskocil I, Tyl J, Kamler M, Titera D, Hakl J, Mrazek J, Bunesova V, Rada V. 2015. Variation in honey bee gut microbial diversity affected by ontogenetic stage, age and geographic location. PLoS One 10:e0118707. doi:10.1371/journal.pone.0118707 PMC4358834 25768309

[10] Ludvigsen J, Porcellato D, L’Abée-Lund TM, Amdam GV, Rudi K. 2017. Geographically widespread honeybee-gut symbiont subgroups show locally distinct antibiotic-resistant patterns. Mol Ecol 26:6590–6607. doi:10.1111/mec.14392 29087008

[11] Tian B, Fadhil NH, Powell JE, Kwong WK, Moran NA. 2012. Long-term exposure to antibiotics has caused accumulation of resistance determinants in the gut microbiota of honeybees. mBio 3:e00377-12. doi:10.1128/mBio.00377-12 PMC3487773 23111871

[12] Baffoni L, Alberoni D, Gaggìa F, Braglia C, Stanton C, Ross PR, Di Gioia D. 2021. Honeybee exposure to veterinary drugs: how is the gut microbiota affected? Microbiol Spectr 9:e0017621. doi:10.1128/spectrum.00176-21 PMC8552759 34378962

[13] Levy SB, Marshall BM. 2013. Honeybees and tetracycline resistance. mBio 4:e00045-13. doi:10.1128/mBio.00045-13 PMC3573660 23404397

[14] Piva S, Giacometti F, Marti E, Massella E, Cabbri R, Galuppi R, Serraino A. 2020. Could honey bees signal the spread of antimicrobial resistance in the environment? Lett Appl Microbiol 70:349–355. doi:10.1111/lam.13288 32096241

[15] Cilia G, Resci I, Scarpellini R, Zavatta L, Albertazzi S, Bortolotti L, Nanetti A, Piva S. 2023. Antimicrobial-resistant environmental bacteria isolated using a network of honey bee colonies (*Apis mellifera* L. 1758). Transbound Emerg Dis 2023:5540574. doi:10.1155/2023/5540574 PMC12016942 40303743

[16] Ludvigsen J, Amdam GV, Rudi K, L’Abée-Lund TM. 2018. Detection and characterization of streptomycin resistance (*strA-strB*) in a honeybee gut symbiont (*Snodgrassella alvi*) and the associated risk of antibiotic resistance transfer. Microb Ecol 76:588–591. doi:10.1007/s00248-018-1171-7 29520453

[17] Stockwell VO, Duffy B. 2012. Use of antibiotics in plant agriculture. Rev Sci Tech 31:199–210. doi:10.20506/rst.31.1.2104 22849276

[18] Okamoto M, Kumagai M, Kanamori H, Takamatsu D. 2021. Antimicrobial resistance genes in bacteria isolated from Japanese honey, and their potential for conferring macrolide and lincosamide resistance in the American foulbrood pathogen *Paenibacillus larvae*. Front Microbiol 12:667096. doi:10.3389/fmicb.2021.667096 PMC8116656 33995331

[19] Sun H, Mu X, Zhang K, Lang H, Su Q, Li X, Zhou X, Zhang X, Zheng H. 2022. Geographical resistome profiling in the honeybee microbiome reveals resistance gene transfer conferred by mobilizable plasmids. Microbiome 10:69. doi:10.1186/s40168-022-01268-1 PMC9063374 35501925

[20] Saccà ML, Resci I, Cilia G. 2024. Phenotypic and genotypic antimicrobial resistance patterns in honey bee (*Apis mellifera* L.) bacterial symbionts. Environ Sci Pollut Res. doi:10.1007/s11356-024-34598-8 39098972

[21] Cenci-Goga BT, Sechi P, Karama M, Ciavarella R, Pipistrelli MV, Goretti E, Elia AC, Gardi T, Pallottini M, Rossi R, Selvaggi R, Grispoldi L. 2020. Cross-sectional study to identify risk factors associated with the occurrence of antimicrobial resistance genes in honey bees *Apis mellifera*) in Umbria, central Italy. Environ Sci Pollut Res 27:9637–9645. doi:10.1007/s11356-020-07629-3 31925681

[22] Resci I, Cilia G. 2023. The use of honey bee (*Apis mellifera* L.) as biological monitors for pathogenic bacteria and antimicrobial resistance: a systematic review. Environ Pollut 333:122120. doi:10.1016/j.envpol.2023.122120 37385360

[23] Nguyen F, Starosta AL, Arenz S, Sohmen D, Dönhöfer A, Wilson DN. 2014. Tetracycline antibiotics and resistance mechanisms. Biol Chem 395:559–575. doi:10.1515/hsz-2013-0292 24497223

[24] Li J, Zhang H, Ning J, Sajid A, Cheng G, Yuan Z, Hao H. 2019. The nature and epidemiology of OqxAB, a multidrug efflux pump. Antimicrob Resist Infect Control 8:44. doi:10.1186/s13756-019-0489-3 PMC6387526 30834112

[25] Mosleh MN, Gharibi M, Alikhani MY, Saidijam M, Vakhshiteh F. 2014. Antimicrobial susceptibility and analysis of macrolide resistance genes in *Streptococcus pneumoniae* isolated in Hamadan. Iran J Basic Med Sci 17:595–599. PMC4240794 25422753

[26] Murphy E. 1985. Nucleotide sequence of ermA, a macrolide-lincosamide-streptogramin B determinant in *Staphylococcus aureus*. J Bacteriol 162:633–640. doi:10.1128/jb.162.2.633-640.1985 PMC218896 2985541

[27] Zhou N, Wei R, Yang S, Hu F, Feng Y, Zheng H. 2026. Antibiotic resistance gene profiles in the gut microbiomes of *Apis cerana*, *Apis mellifera*, and *Bombus terrestris*. Pestic Biochem Physiol 220:107059. doi:10.1016/j.pestbp.2026.107059 42034426

[28] Lawther K, Santos FG, Oyama LB, Rubino F, Morrison S, Creevey CJ, McGrath JW, Huws SA. 2022. Resistome analysis of global livestock and soil microbiomes. Front Microbiol 13:897905. doi:10.3389/fmicb.2022.897905 PMC9300982 35875563

[29] Kulková N, Babálová M, Brnová J, Krcméry V. 2014. Transferable fluoroquinolone resistance in *Enterobacteriaceae* and *Pseudomonas aeruginosa* isolated from hemocultures. Cent Eur J Public Health 22:60–63. doi:10.21101/cejph.a3884 24844110

[30] Sun J, Liao X-P, D’Souza AW, Boolchandani M, Li S-H, Cheng K, Luis Martínez J, Li L, Feng Y-J, Fang L-X, et al.. 2020. Environmental remodeling of human gut microbiota and antibiotic resistome in livestock farms. Nat Commun 11:1427. doi:10.1038/s41467-020-15222-y PMC7080799 32188862

[31] MacGowan A, Macnaughton E. 2017. Antibiotic resistance. Medicine 45:622–628. doi:10.1016/j.mpmed.2017.07.006

[32] Peck DT, Seeley TD. 2019. Mite bombs or robber lures? The roles of drifting and robbing in *Varroa destructor* transmission from collapsing honey bee colonies to their neighbors. PLoS One 14:e0218392. doi:10.1371/journal.pone.0218392 PMC6588223 31226130

[33] Copeland DC, Anderson KE, Mott BM. 2022. Early queen development in honey bees: social context and queen breeder source affect gut microbiota and associated metabolism. Microbiol Spectr 10:e0038322. doi:10.1128/spectrum.00383-22 PMC9430896 35867384

[34] Butler CG. 1940. The choice of drinking water by the honeybee. J Exp Biol 17:253–261. doi:10.1242/jeb.17.3.253

[35] Fry KL, McPherson VJ, Gillings MR, Taylor MP. 2023. Tracing the sources and prevalence of class 1 integrons, antimicrobial resistance, and trace elements using European honey bees. Environ Sci Technol 57:10582–10590. doi:10.1021/acs.est.3c03775 37417314

[36] Johnson KB, Smith TJ, Temple TN, Gutierrez E, Elkins RB, Castagnoli SP. 2016. Integration of acibenzolar-S-methyl with antibiotics for protection of pear and apple from fire blight caused by *Erwinia amylovora*. Crop Protection 88:149–154. doi:10.1016/j.cropro.2016.06.010

[37] Khan KA, Liu T. 2022. Morphological structure and distribution of hairiness on different body parts of *Apis mellifera* with an implication on pollination biology and a novel method to measure the hair length. Insects 13:189. doi:10.3390/insects13020189 PMC8874558 35206762

[38] Woodford N, Ellington MJ. 2007. The emergence of antibiotic resistance by mutation. Clin Microbiol Infect 13:5–18. doi:10.1111/j.1469-0691.2006.01492.x 17184282

[39] Knöppel A, Näsvall J, Andersson DI. 2017. Evolution of antibiotic resistance without antibiotic exposure. Antimicrob Agents Chemother 61:e01495-17. doi:10.1128/AAC.01495-17 PMC5655081 28893783

[40] Bottery MJ, Pitchford JW, Friman V-P. 2021. Ecology and evolution of antimicrobial resistance in bacterial communities. ISME J 15:939–948. doi:10.1038/s41396-020-00832-7 PMC8115348 33219299

[41] Engel P, Kwong WK, Moran NA. 2013. *Frischella perrara* gen. nov., sp. nov., a gammaproteobacterium isolated from the gut of the honeybee, *Apis mellifera*. Int J Syst Evol Microbiol 63:3646–3651. doi:10.1099/ijs.0.049569-0 23606484

[42] Bolotin E, Hershberg R. 2017. Horizontally acquired genes are often shared between closely related bacterial species. Front Microbiol 8:1536. doi:10.3389/fmicb.2017.01536 PMC5575156 28890711

[43] Qin Z, Gao Q, Dong Q, Van Nostrand JD, Qi Q, Su Y, Liu S, Dai T, Cheng J, Zhou J, Yang Y. 2022. Antibiotic resistome mostly relates to bacterial taxonomy along a suburban transmission chain. Front Environ Sci Eng 16:32. doi:10.1007/s11783-021-1466-7

[44] Qu J, Wu Y, Liu Y, Cui Y, Zhao M, Zhu H, Zhang Q. 2021. Metagenomics reveals the taxonomy and resistance mechanism of antibiotic resistance genes in bacterial communities of an aquaculture pond. J Phys: Conf Ser 2009:012032. doi:10.1088/1742-6596/2009/1/012032

[45] Kim S, Michaels BD, Kim GK, Del Rosso JQ. 2013. 8 - Systemic antibacterial agents, p 61–97. In Wolverton SE (ed), Comprehensive dermatologic drug therapy, Third Edition. W.B. Saunders, London.

[46] Casacuberta E, González J. 2013. The impact of transposable elements in environmental adaptation. Mol Ecol 22:1503–1517. doi:10.1111/mec.12170 23293987

[47] Huang H, Wei L, Li L, He Z, Zhang K, Bao M, Sun W, Li X, Zhang J, Yu P, Wang J, Chen Y. 2025. Microbial interactions as the key to understanding and controlling environmental spread of antibiotic resistance genes. NPJ Antimicrob Resist 3:97. doi:10.1038/s44259-025-00174-4 PMC12678825 41345232

[48] Quigley TP, Amdam GV, Harwood GH. 2019. Honey bees as bioindicators of changing global agricultural landscapes. Curr Opin Insect Sci 35:132–137. doi:10.1016/j.cois.2019.08.012 31541967

[49] Laconi A, Tolosi R, Mughini-Gras L, Mazzucato M, Ferrè N, Carraro L, Cardazzo B, Capolongo F, Merlanti R, Piccirillo A. 2022. Beehive products as bioindicators of antimicrobial resistance contamination in the environment. Sci Total Environ 823:151131. doi:10.1016/j.scitotenv.2021.151131 34695463

[50] Bell WJ. 1990. Central place foraging, p 171–187. In Bell WJ (ed), Searching behaviour: the behavioural ecology of finding resources. Springer Netherlands, Dordrecht.

[51] Koch H, Schmid-Hempel P. 2011. Bacterial communities in central European bumblebees: low diversity and high specificity. Microb Ecol 62:121–133. doi:10.1007/s00248-011-9854-3 21556885

[52] Engel P, James RR, Koga R, Kwong WK, McFrederick QS, Moran NA. 2013. Standard methods for research on *Apis mellifera* gut symbionts. J Apic Res 52:1–24. doi:10.3896/IBRA.1.52.4.07

[53] O’Leary NA, Wright MW, Brister JR, Ciufo S, Haddad D, McVeigh R, Rajput B, Robbertse B, Smith-White B, Ako-Adjei D, et al.. 2016. Reference sequence (RefSeq) database at NCBI: current status, taxonomic expansion, and functional annotation. Nucleic Acids Res 44:D733–45. doi:10.1093/nar/gkv1189 PMC4702849 26553804

[54] Li H. 2013. Aligning sequence reads, clone sequences and assembly contigs with BWA-MEM. arXiv:1303–3997. doi:10.48550/arXiv.1303.3997

[55] Danecek P, Bonfield JK, Liddle J, Marshall J, Ohan V, Pollard MO, Whitwham A, Keane T, McCarthy SA, Davies RM, Li H. 2021. Twelve years of SAMtools and BCFtools. Gigascience 10:giab008. doi:10.1093/gigascience/giab008 PMC7931819 33590861

[56] Tamames J, Puente-Sánchez F. 2018. SqueezeMeta, a highly portable, fully automatic metagenomic analysis pipeline. Front Microbiol 9:3349. doi:10.3389/fmicb.2018.03349 PMC6353838 30733714

[57] Aminov RI, Chee-Sanford JC, Garrigues N, Mehboob A, Mackie RI. 2004. Detection of tetracycline resistance genes by PCR methods, p 3–13. In Spencer JFT, Spencer AL (ed), Public health microbiology: methods and protocols. Humana Press, Totowa, NJ. 10.1385/1-59259-766-1:003 15156014

[58] Aminov RI, Chee-Sanford JC, Garrigues N, Teferedegne B, Krapac IJ, White BA, Mackie RI. 2002. Development, validation, and application of PCR primers for detection of tetracycline efflux genes of gram-negative bacteria. Appl Environ Microbiol 68:1786–1793. doi:10.1128/AEM.68.4.1786-1793.2002 PMC123860 11916697

[59] Aminov RI, Garrigues-Jeanjean N, Mackie RI. 2001. Molecular ecology of tetracycline resistance: development and validation of primers for detection of tetracycline resistance genes encoding ribosomal protection proteins. Appl Environ Microbiol 67:22–32. doi:10.1128/AEM.67.1.22-32.2001 PMC92507 11133424

[60] Seemann T. 2020. Abricate. GitHub. Available from: https://github.com/tseemann/abricate/blob/master/README.md

[61] R Core Team. 2025. R: a language and environment for statistical computing (4.5.0). R Foundation for Statistical Computing, Vienna, Austria.

[62] McMurdie PJ, Holmes S. 2013. Phyloseq: an R package for reproducible interactive analysis and graphics of microbiome census data. PLoS One 8:e61217. doi:10.1371/journal.pone.0061217 PMC3632530 23630581

[63] Dixon P. 2003. VEGAN, a package of R functions for community ecology. J Vegetation Science 14:927–930. doi:10.1111/j.1654-1103.2003.tb02228.x

[64] Hijmans RJ. 2026. Geosphere: spherical trigonometry (R package version 1.6-7). https://github.com/rspatial/geosphere.

[65] Vasimuddin M, Misra S, Li H, Aluru S. 2019. Efficient architecture-aware acceleration of BWA-MEM for multicore systems. 2019 IEEE International Parallel and Distributed Processing Symposium (IPDPS.

